# Supplementary material for: Revisiting the p53:Sirt1 interaction in light of controlling p53 acetylation levels
Source: Commun Chem. 2026 Jul 10;9:243. doi: 10.1038/s42004-026-02127-y (PMC13354560; doi:10.1038/s42004-026-02127-y)
Supplement: Supplementary file 1 — Supplementary Information [file 42004_2026_2127_MOESM1_ESM.pdf]

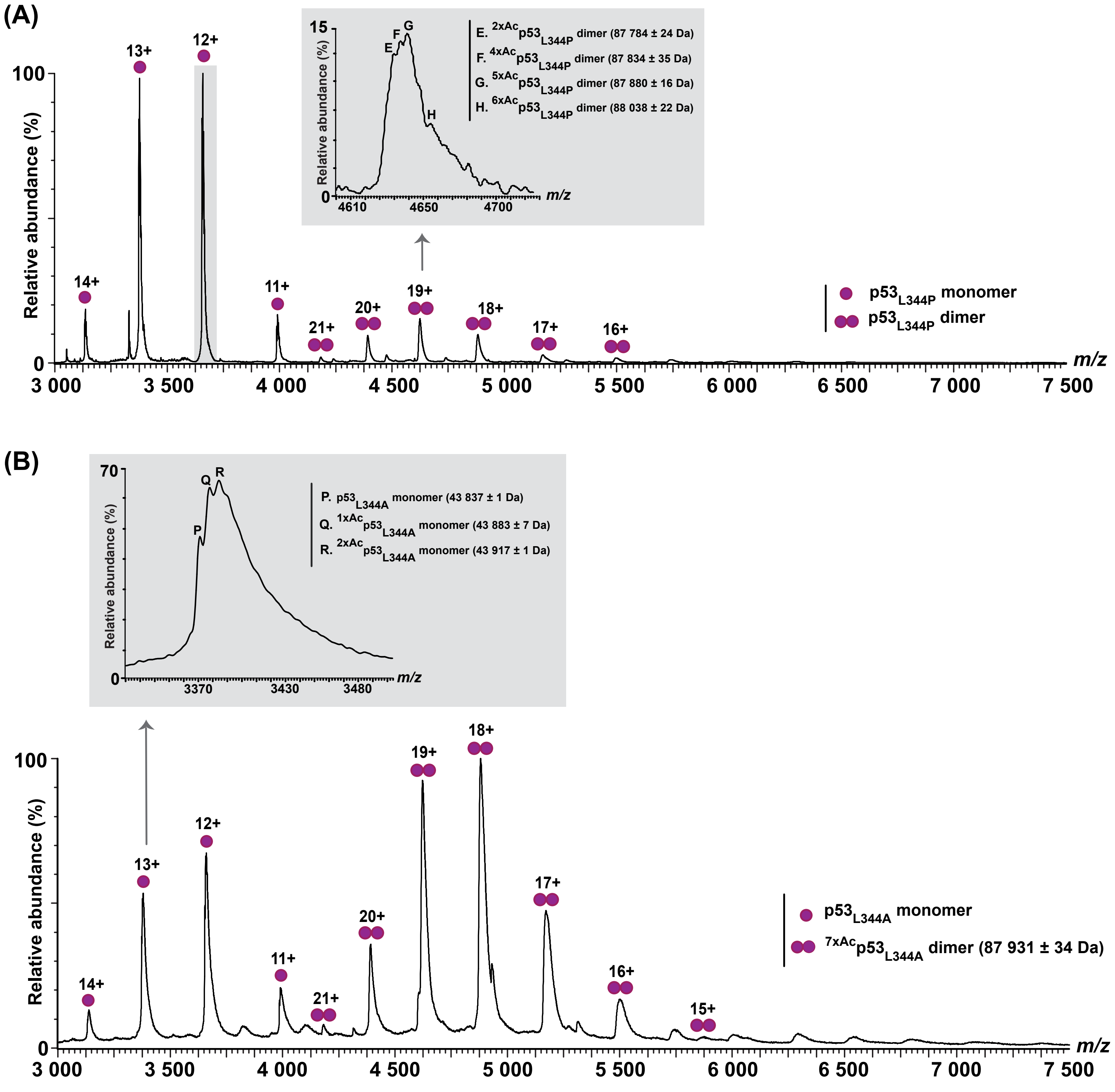
(A)
E. 2xAcp53L344P dimer (87 784 ± 24 Da)
F. 4xAcp53L344P dimer (87 834 ± 35 Da)
G. 5xAcp53L344P dimer (87 880 ± 16 Da)
H. 6xAcp53L344P dimer (88 038 ± 22 Da)
Relative abundance (%)
m/z
14+
13+
12+
11+
21+
20+
19+
18+
17+
16+
p53L344P monomer
p53L344P dimer
(B)
P. p53L344A monomer (43 837 ± 1 Da)
Q. 1xAcp53L344A monomer (43 883 ± 7 Da)
R. 2xAcp53L344A monomer (43 917 ± 1 Da)
15+
p53L344A monomer
7xAcp53L344A dimer (87 931 ± 34 Da)

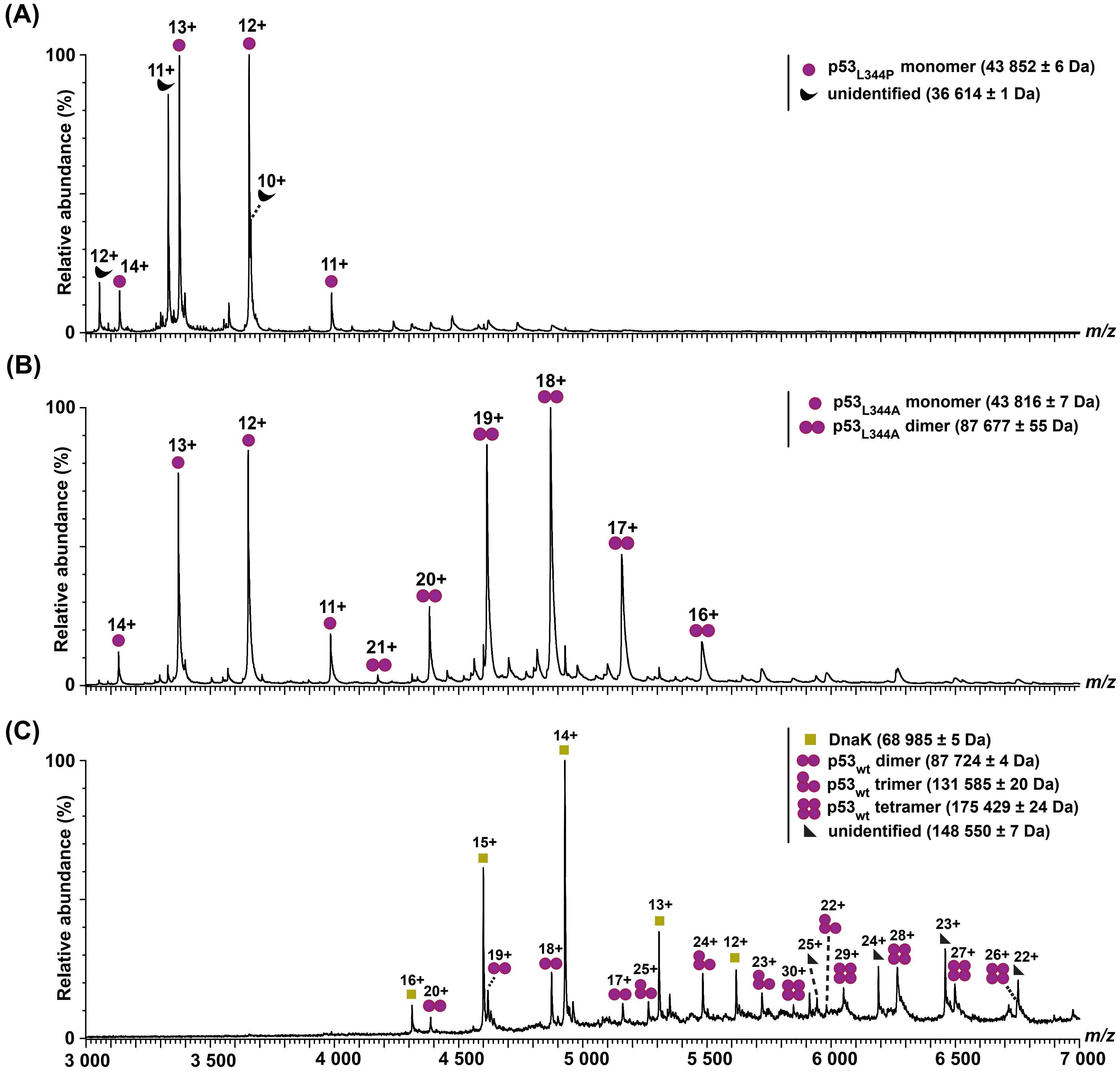

(A)
(B)
(C)
Relative abundance (%)
m/z
p53L344P monomer (43 852 ± 6 Da)
unidentified (36 614 ± 1 Da)
p53L344A monomer (43 816 ± 7 Da)
p53L344A dimer (87 677 ± 55 Da)
DnaK (68 985 ± 5 Da)
p53wt dimer (87 724 ± 4 Da)
p53wt trimer (131 585 ± 20 Da)
p53wt tetramer (175 429 ± 24 Da)
unidentified (148 550 ± 7 Da)
3 000
3 500
4 000
4 500
5 000
5 500
6 000
6 500
7 000

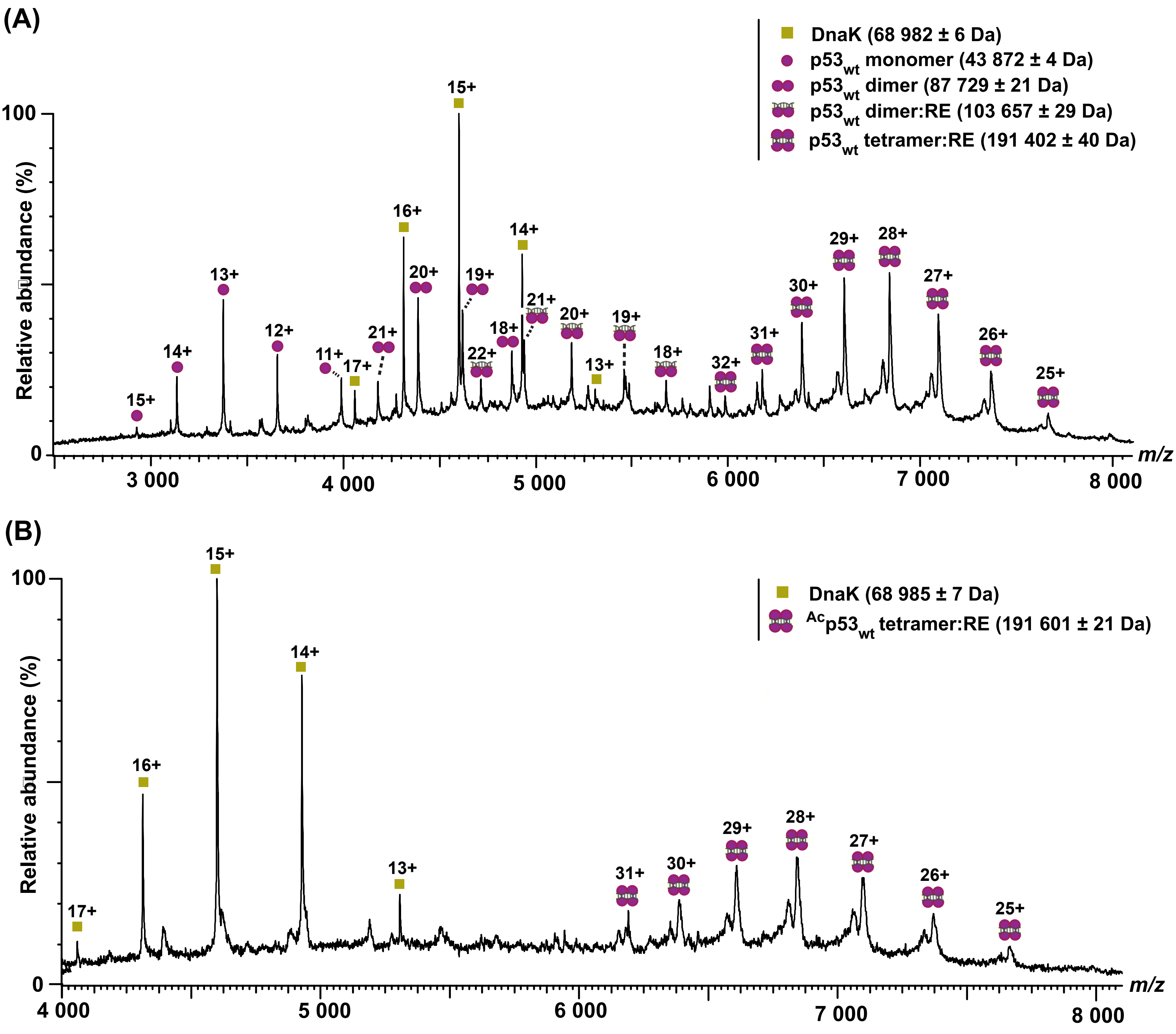
(A)
DnaK (68 982 ± 6 Da)
p53wt monomer (43 872 ± 4 Da)
p53wt dimer (87 729 ± 21 Da)
p53wt dimer:RE (103 657 ± 29 Da)
p53wt tetramer:RE (191 402 ± 40 Da)
Relative abundance (%)
m/z
(B)
DnaK (68 985 ± 7 Da)
Acp53wt tetramer:RE (191 601 ± 21 Da)
Relative abundance (%)
m/z

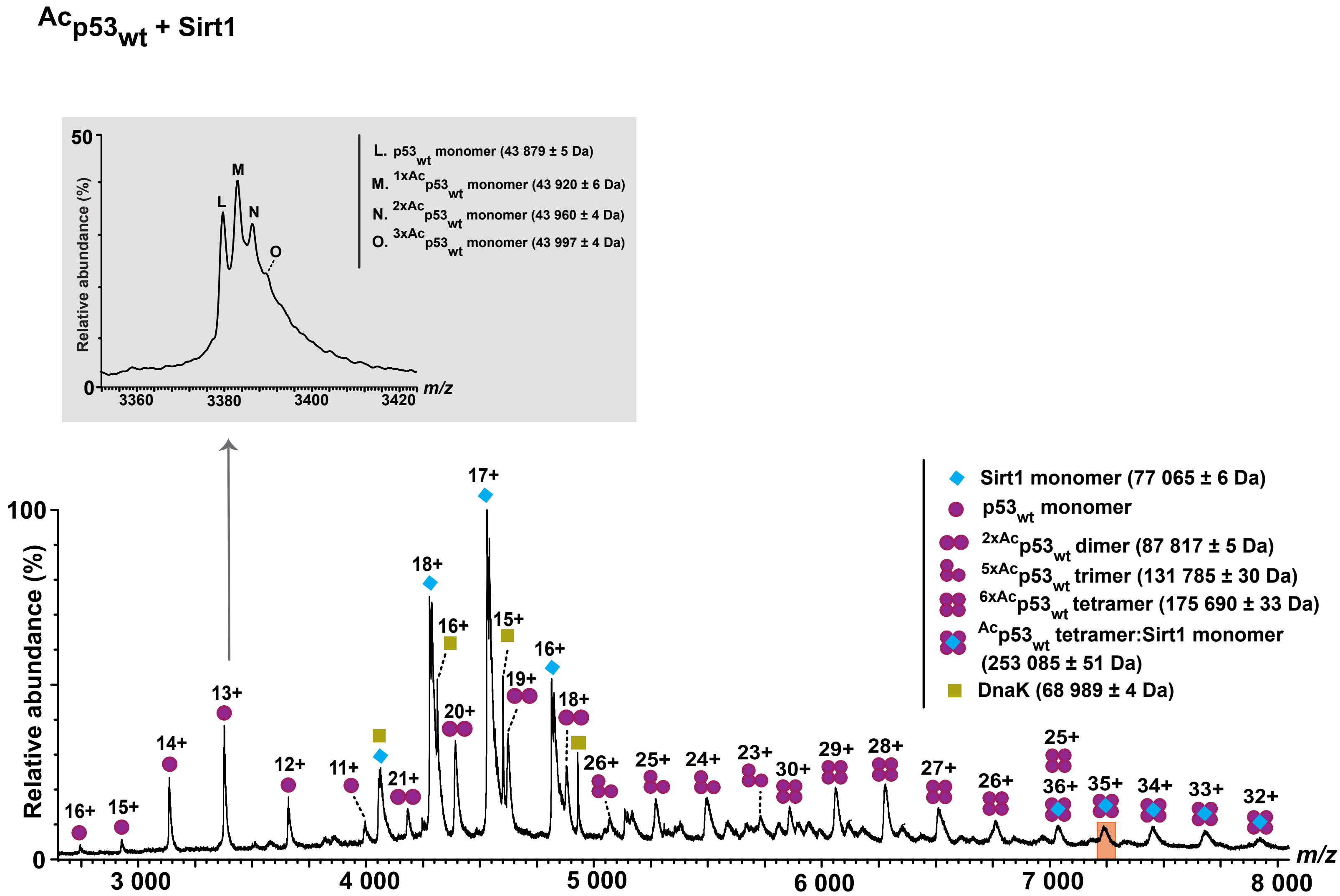

Acp53wt + Sirt1
Relative abundance (%)
m/z
L
M
N
O
L. p53wt monomer (43 879 ± 5 Da)
M. 1xAcp53wt monomer (43 920 ± 6 Da)
N. 2xAcp53wt monomer (43 960 ± 4 Da)
O. 3xAcp53wt monomer (43 997 ± 4 Da)
Sirt1 monomer (77 065 ± 6 Da)
p53wt monomer
2xAcp53wt dimer (87 817 ± 5 Da)
5xAcp53wt trimer (131 785 ± 30 Da)
6xAcp53wt tetramer (175 690 ± 33 Da)
Acp53wt tetramer:Sirt1 monomer (253 085 ± 51 Da)
DnaK (68 989 ± 4 Da)

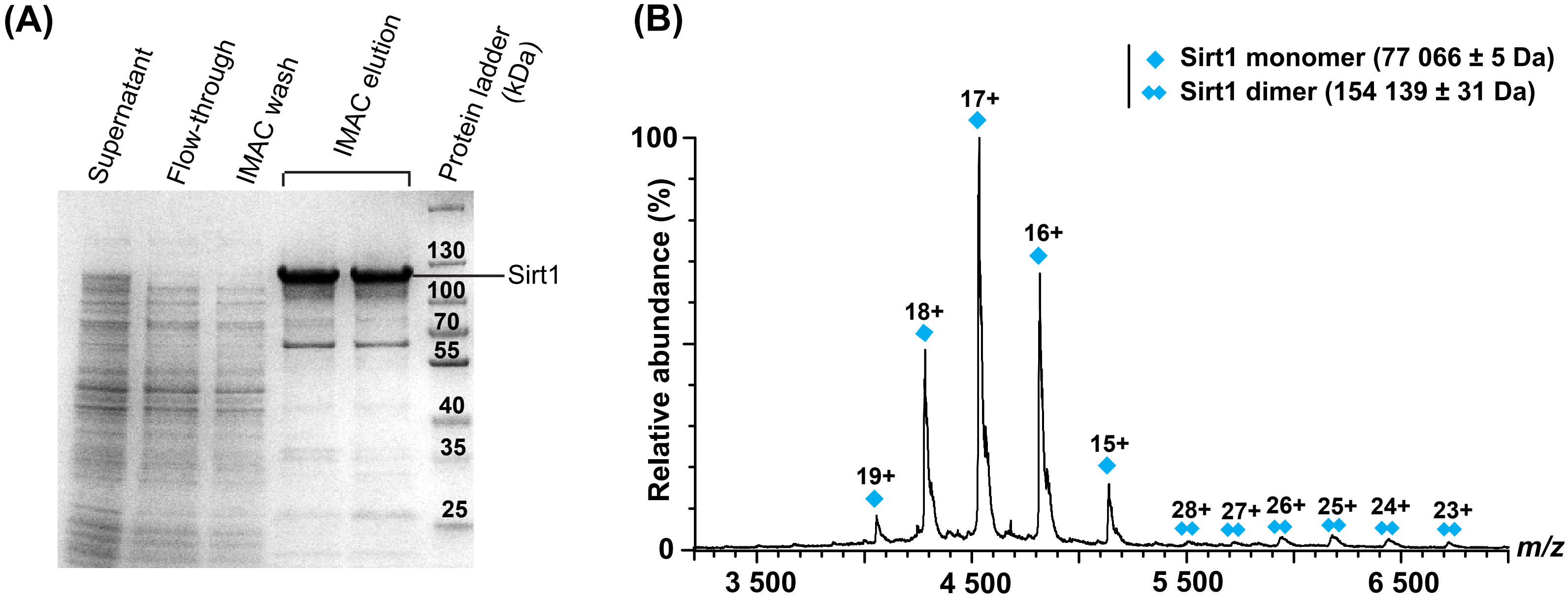

(A)
Supernatant
Flow-through
IMAC wash
IMAC elution
Protein ladder (kDa)
130
100
70
55
40
35
25
Sirt1
(B)
Sirt1 monomer (77 066 ± 5 Da)
Sirt1 dimer (154 139 ± 31 Da)
Relative abundance (%)
100
0
19+
18+
17+
16+
15+
28+
27+
26+
25+
24+
23+
m/z
3 500
4 500
5 500
6 500

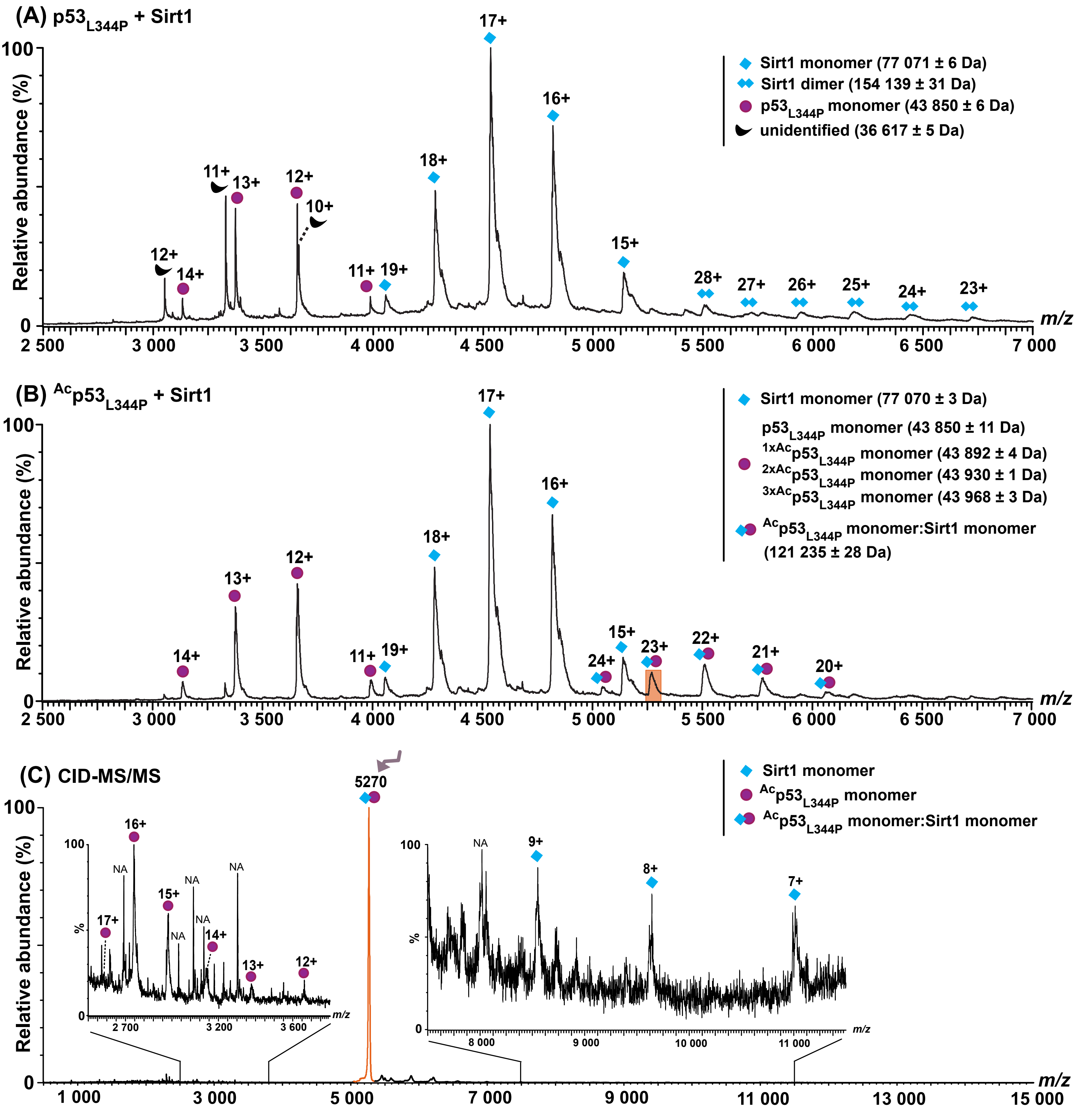

(A) p53L344P + Sirt1
Sirt1 monomer (77 071 ± 6 Da)
Sirt1 dimer (154 139 ± 31 Da)
p53L344P monomer (43 850 ± 6 Da)
unidentified (36 617 ± 5 Da)
Relative abundance (%)
m/z
(B) Acp53L344P + Sirt1
Sirt1 monomer (77 070 ± 3 Da)
p53L344P monomer (43 850 ± 11 Da)
1xAcp53L344P monomer (43 892 ± 4 Da)
2xAcp53L344P monomer (43 930 ± 1 Da)
3xAcp53L344P monomer (43 968 ± 3 Da)
Acp53L344P monomer:Sirt1 monomer
(121 235 ± 28 Da)
(C) CID-MS/MS
5270
Sirt1 monomer
Acp53L344P monomer
Acp53L344P monomer:Sirt1 monomer
NA

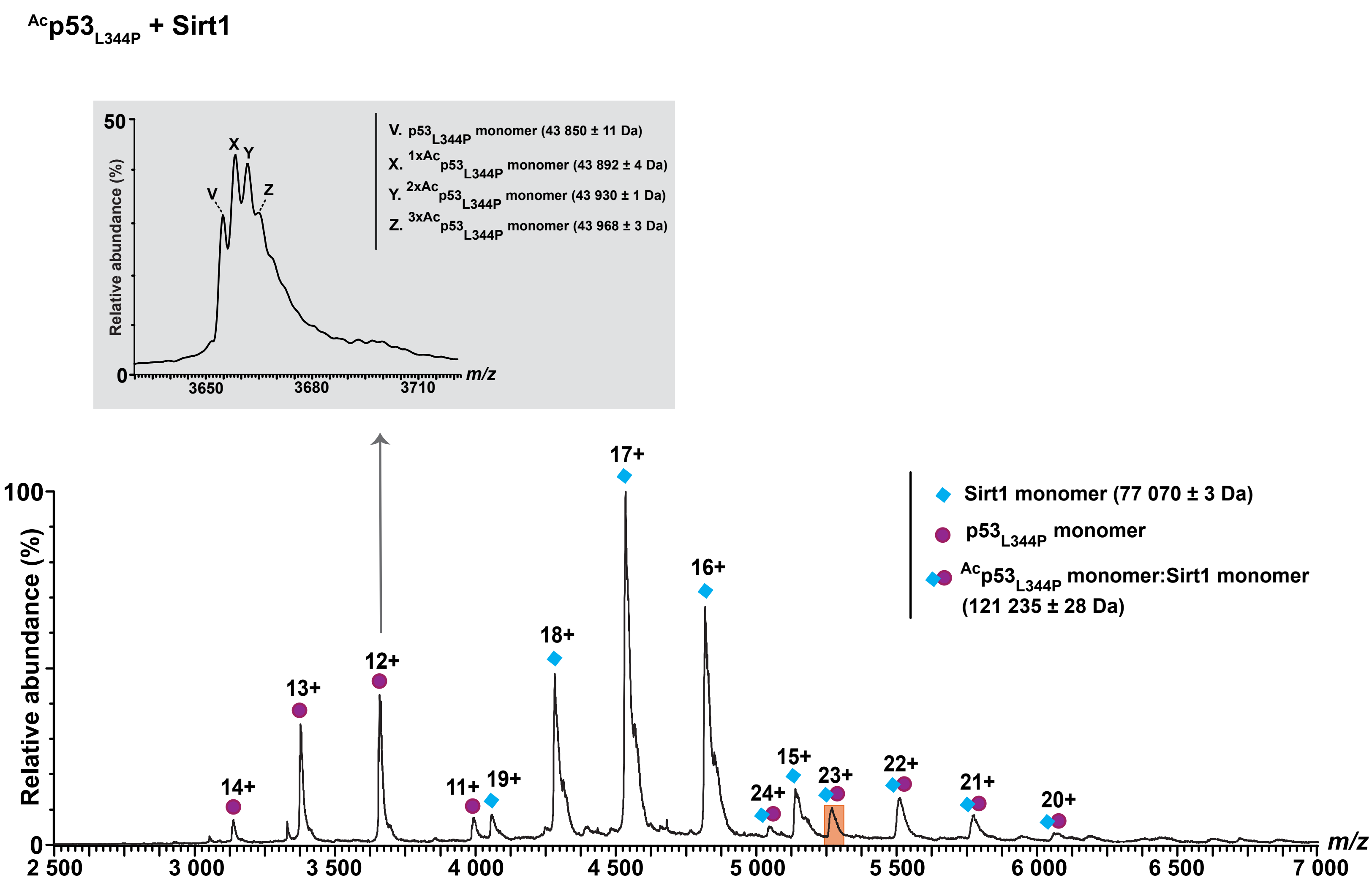

$^{Ac}p53_{L344P}$ + Sirt1
50
0
Relative abundance (%)
V
X
Y
Z
3650
3680
3710
m/z
V. $p53_{L344P}$ monomer (43 850 ± 11 Da)
X. $^{1xAc}p53_{L344P}$ monomer (43 892 ± 4 Da)
Y. $^{2xAc}p53_{L344P}$ monomer (43 930 ± 1 Da)
Z. $^{3xAc}p53_{L344P}$ monomer (43 968 ± 3 Da)
100
0
Relative abundance (%)
14+
13+
12+
11+
19+
18+
17+
16+
24+
15+
23+
22+
21+
20+
2 500
3 000
3 500
4 000
4 500
5 000
5 500
6 000
6 500
7 000
m/z
Sirt1 monomer (77 070 ± 3 Da)
$p53_{L344P}$ monomer
$^{Ac}p53_{L344P}$ monomer:Sirt1 monomer
(121 235 ± 28 Da)

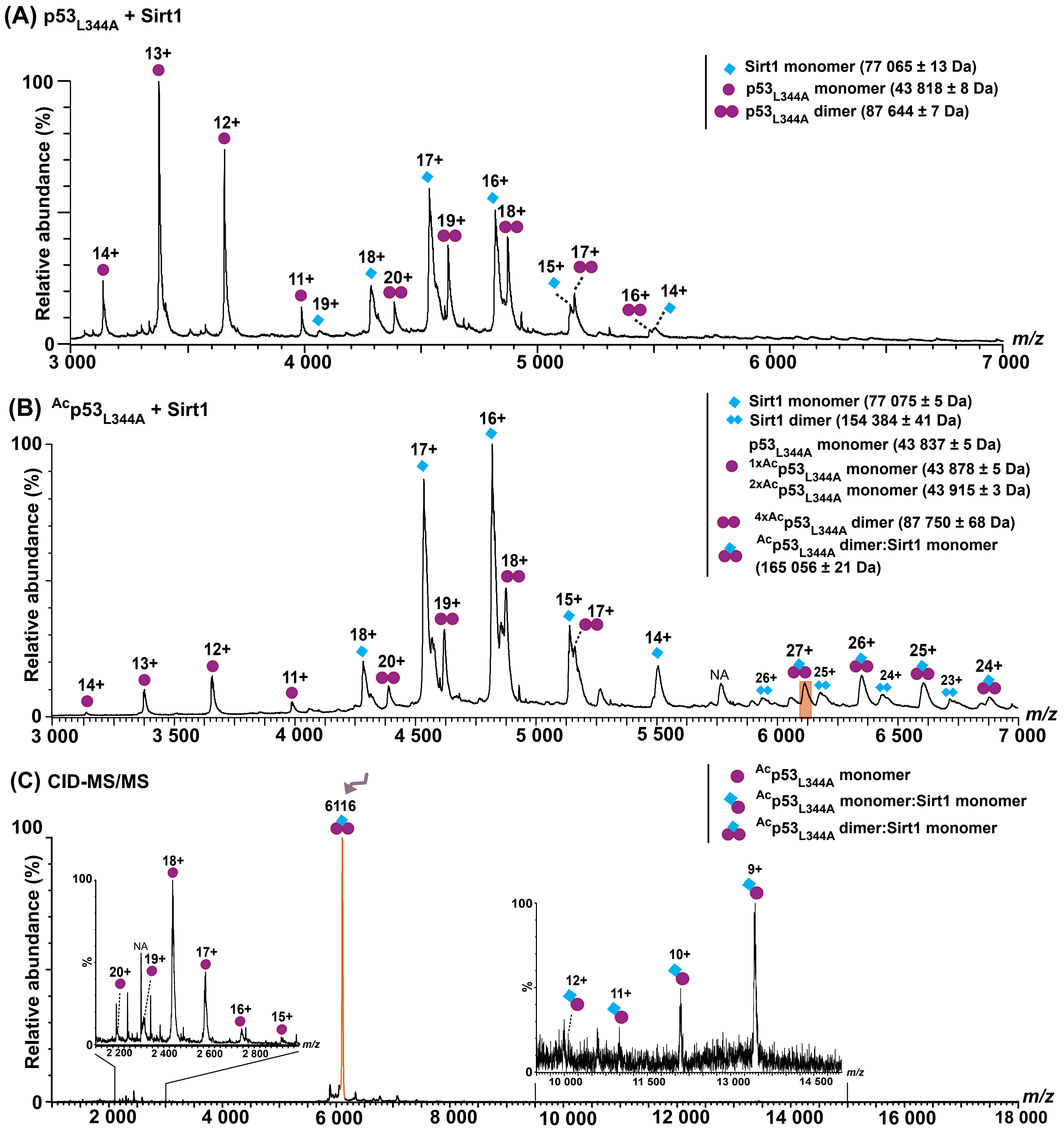
(A) p53L344A + Sirt1
Sirt1 monomer (77 065 ± 13 Da)
p53L344A monomer (43 818 ± 8 Da)
p53L344A dimer (87 644 ± 7 Da)
Relative abundance (%)
m/z
(B) Acp53L344A + Sirt1
Sirt1 monomer (77 075 ± 5 Da)
Sirt1 dimer (154 384 ± 41 Da)
p53L344A monomer (43 837 ± 5 Da)
1xAcp53L344A monomer (43 878 ± 5 Da)
2xAcp53L344A monomer (43 915 ± 3 Da)
4xAcp53L344A dimer (87 750 ± 68 Da)
Acp53L344A dimer:Sirt1 monomer (165 056 ± 21 Da)
NA
(C) CID-MS/MS
Acp53L344A monomer
Acp53L344A monomer:Sirt1 monomer
Acp53L344A dimer:Sirt1 monomer
6116

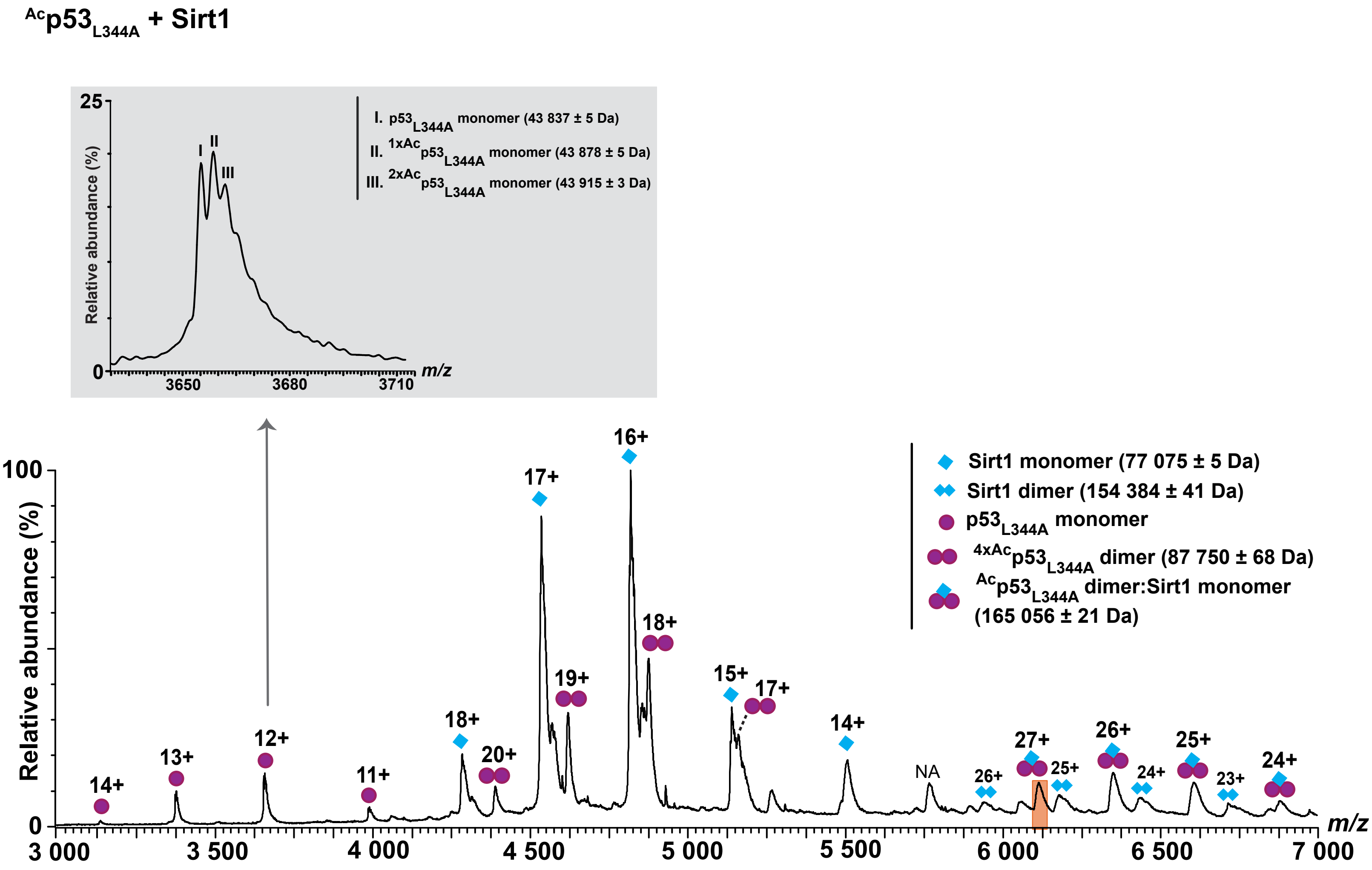

Acp53L344A + Sirt1
I. p53L344A monomer (43 837 ± 5 Da)
II. 1xAcp53L344A monomer (43 878 ± 5 Da)
III. 2xAcp53L344A monomer (43 915 ± 3 Da)
Relative abundance (%)
m/z
Sirt1 monomer (77 075 ± 5 Da)
Sirt1 dimer (154 384 ± 41 Da)
p53L344A monomer
4xAcp53L344A dimer (87 750 ± 68 Da)
Acp53L344A dimer:Sirt1 monomer (165 056 ± 21 Da)
NA

a)

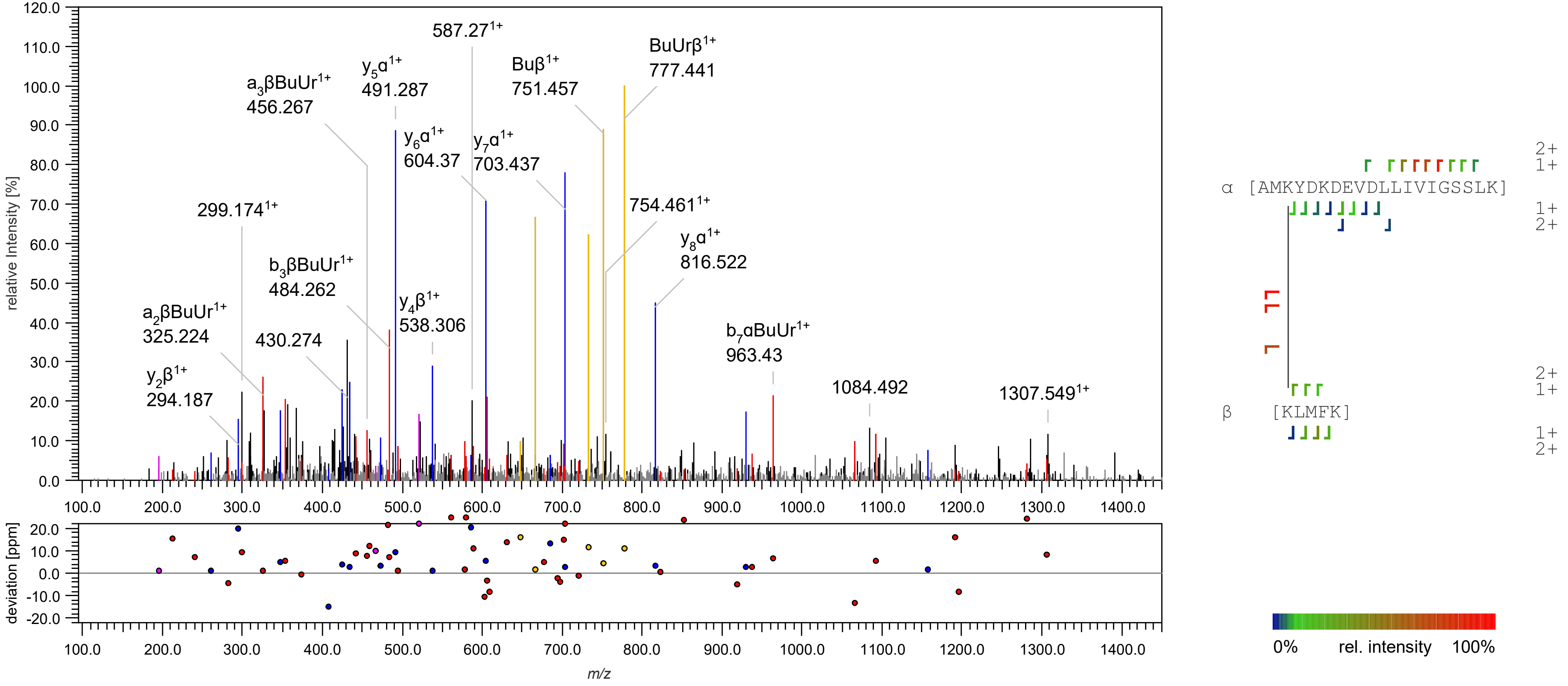

120.0
110.0
100.0
90.0
80.0
70.0
60.0
50.0
40.0
30.0
20.0
10.0
0.0
relative Intensity [%]
587.27^{1+}
BuUrβ^{1+} 777.441
Buβ^{1+} 751.457
$y_5α^{1+}$ 491.287
$a_3βBuUr^{1+}$ 456.267
$y_6α^{1+}$ 604.37
$y_7α^{1+}$ 703.437
299.174^{1+}
754.461^{1+}
$y_8α^{1+}$ 816.522
$b_3βBuUr^{1+}$ 484.262
$y_4β^{1+}$ 538.306
$a_2βBuUr^{1+}$ 325.224
430.274
$b_7αBuUr^{1+}$ 963.43
$y_2β^{1+}$ 294.187
1084.492
1307.549^{1+}
100.0 200.0 300.0 400.0 500.0 600.0 700.0 800.0 900.0 1000.0 1100.0 1200.0 1300.0 1400.0
20.0
10.0
0.0
-10.0
-20.0
deviation [ppm]
m/z
α [AMKYDKDEVDLLIVIGSSLK]
β [KLMFK]
2+
1+
0%
rel. intensity
100%

b)

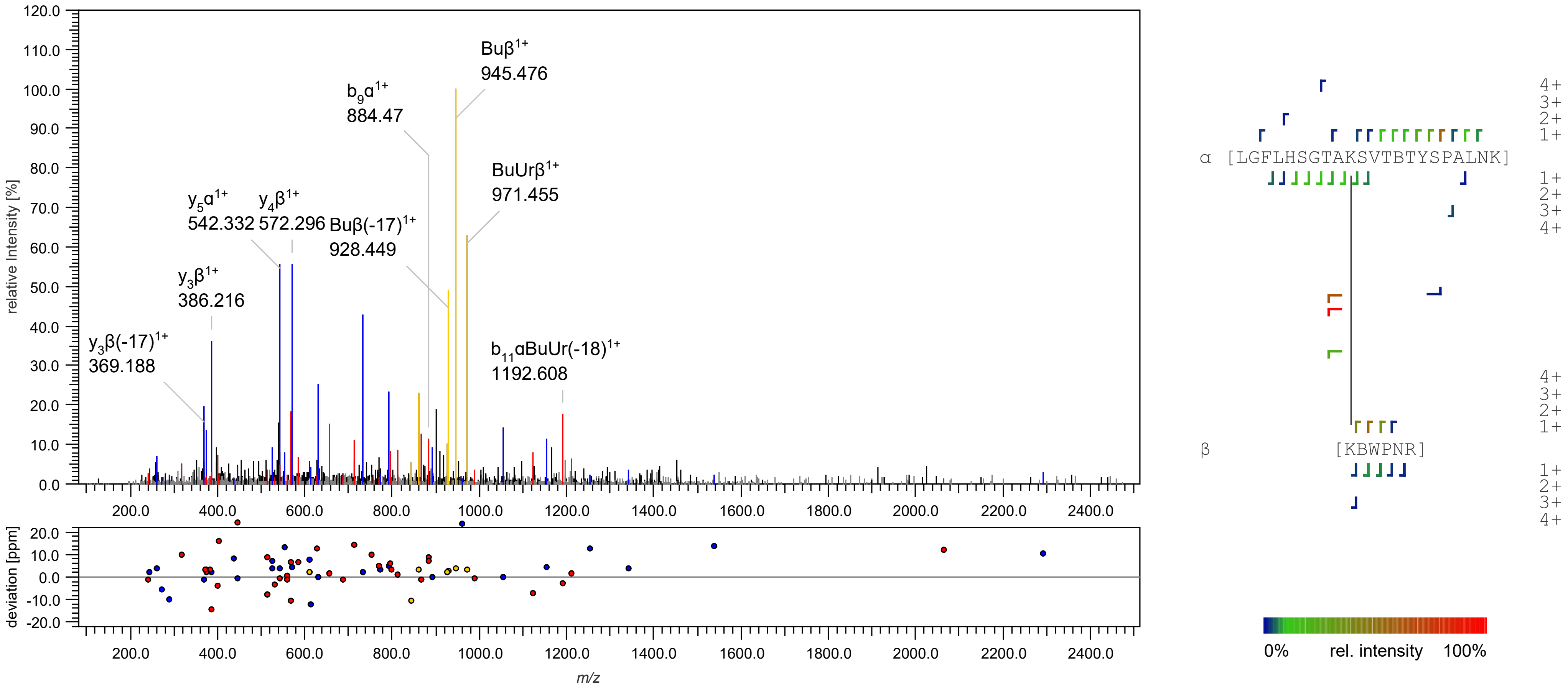

Buβ1+ 945.476
b9α1+ 884.47
BuUrβ1+ 971.455
y5α1+ 542.332
y4β1+ 572.296
Buβ(-17)1+ 928.449
y3β1+ 386.216
y3β(-17)1+ 369.188
b11αBuUr(-18)1+ 1192.608
relative Intensity [%]
deviation [ppm]
m/z
α [LGFLHSGTAKSVTBTYSPALNK]
β [KBWPNR]
1+
2+
3+
4+
0%
rel. intensity
100%

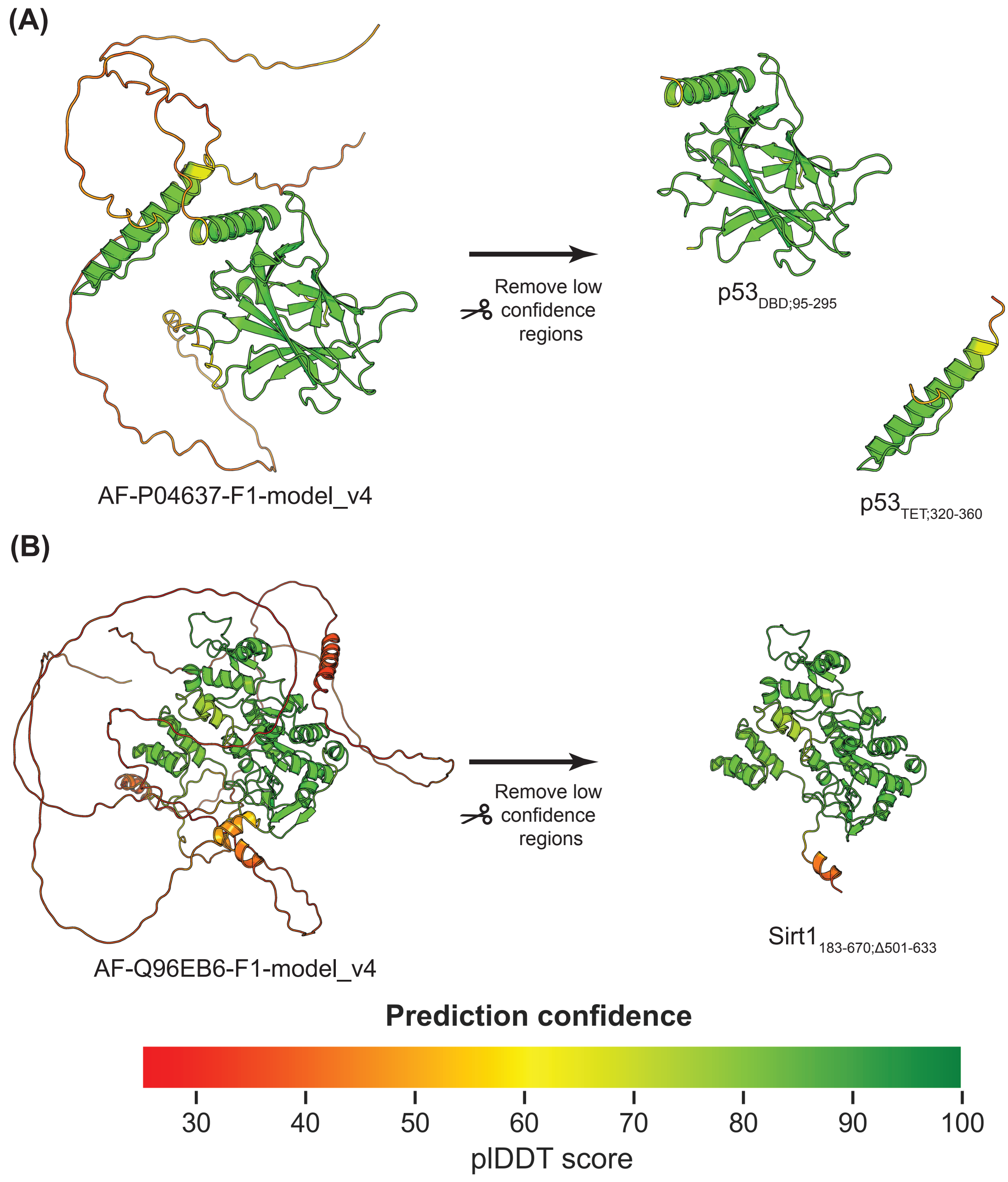

(A)
Remove low confidence regions
p53 DBD;95-295
p53 TET;320-360
AF-P04637-F1-model_v4
(B)
Remove low confidence regions
Sirt1 183-670;Δ501-633
AF-Q96EB6-F1-model_v4
Prediction confidence
30
40
50
60
70
80
90
100
plDDT score

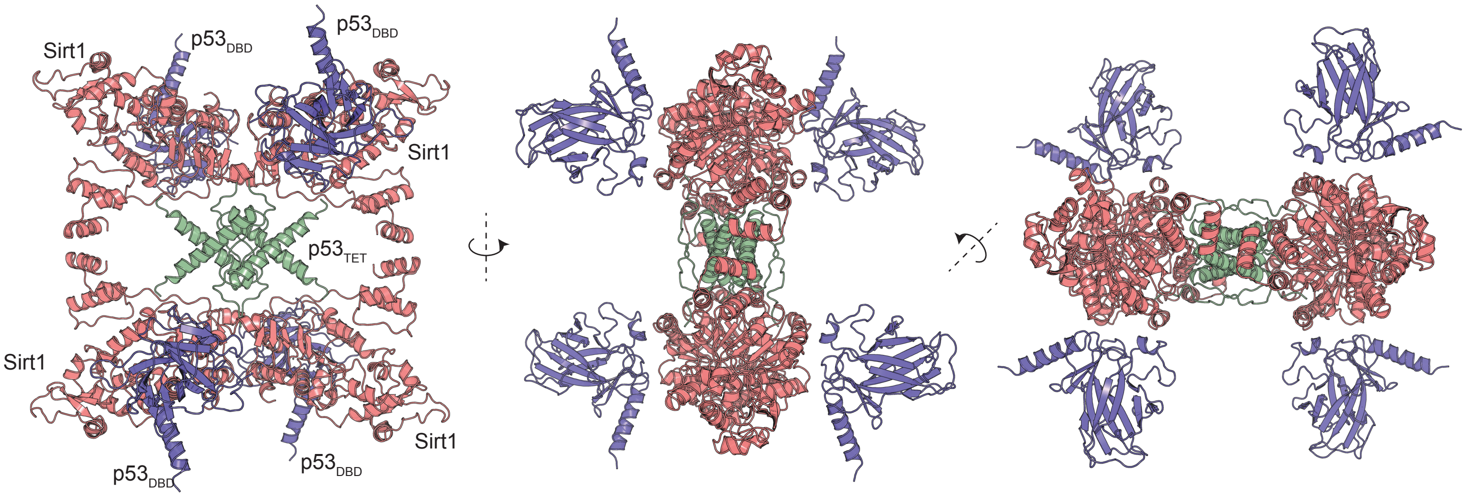

Sirt1
p53$_{DBD}$
p53$_{DBD}$
Sirt1
p53$_{TET}$
Sirt1
Sirt1
p53$_{DBD}$
p53$_{DBD}$

Figure S1: Native mass spectra of acetylated full-length human p53 expressed in *E. coli*. Each p53 monomer is shown as a purple circle, DnaK is shown as yellow square. (A) $^{Ac}p53_{L344P}$ forms monomers (charge states 11+ to 14+). Also, dimers are formed with two, four, five, and six acetylations (charge states 16+ to 21+). (B) $^{Ac}p53_{L344A}$ forms monomers (charge states 11+ to 14+) with zero, one, and two acetylations as well as dimers (charge states 15+ to 21+) with seven acetylations.

Figure S2: Native mass spectra of purified non-acetylated p53 variants. (A) $p53_{L344P}$. forms monomers (charge states 11+ to 14+). (B) $p53_{L344A}$ forms monomers (charge states 11+ to 14+) and dimers (charge states 16+ to 21+). (C) $p53_{wt}$. forms dimers (charge states 17+ to 20+), trimers (charge states 23+ to 25+), and tetramers (charge states 26+ to 30+).

Figure S3: Native mass spectra of DNA response element (RE)-bound $p53_{wt}$. (A) Non-acetylated $p53_{wt}$ forms monomers (charge states 11+ to 15+) and dimers (charge states 18+ to 21+). In addtion, DNA RE-bound $p53_{wt}$ dimers (charge states 18+ to 22+) and tetramers (charge states 25+ to 32+) were observed. (B) $^{Ac}p53_{wt}$ forms DNA RE-bound tetramer (charge states 25+ to 31+).

Figure S4: Native MS confirms acetylation-dependent complex formation between $p53_{wt}$ and Sirt1. For $^{Ac}p53_{wt}$, Sirt1 monomer (charge states 16+ to 19+) as well as p53 monomer (charge states 11+ to 16+ with zero, one, two, or three acetylations), dimer (charge states 18+ to 21+), trimer (charge states 23+ to 26+), tetramer (charge states 25+ to 30+) are observed. Additionally, the $^{Ac}p53_{wt}$:Sirt1 complex was detected (charge states 32+ to 36+).

Figure S5: SDS-PAGE and native MS of Sirt1. (A) SDS-PAGE of samples collected at different stages of Sirt1 purification. (B) Native mass spectrum depicting purified intact Sirt1 (blue diamonds) in monomeric (charge states 15+ to 19+) and dimeric (charge states 23+ to 28+) state.

Figure S6: Native MS confirms acetylation-dependent complex formation between $p53_{L344P}$ and Sirt1. (A) For non-acetylated $p53_{L344P}$, Sirt1 monomer (charge states 15+ to 20+) and dimer (charge states 23+ to 28+) as well as $p53_{L344P}$ monomer (charge states 12+ to 15+) was observed. (B) For $^{Ac}p53_{L344P}$, Sirt1 monomer (charge states 16+ to 20+) and dimer (charge states 24+ to 28+), p53 monomer (charge states 12+ to 15+), and the $^{Ac}p53_{L344P}$:Sirt1 complex (1:1) (charge states 21+ to 24+) have been detected. (C) Collisional activation (CID-MS/MS) of the 23+ charge state of this complex (orange box in B) resulted in the ejection of a $^{Ac}p53_{L344P}$ monomer (charge states 12+ to 17+, m/z ~2 600 to ~3 700) from the complex. The remaining Sirt1 (charge states 7+ to 9+, m/z ~7 500 to ~11 000) was observed as well.

Figure S7: Native MS confirms acetylation-dependent complex formation between $p53_{L344P}$ and Sirt1. For $^{Ac}p53_{L344P}$, Sirt1 monomer (charge states 16+ to 20+) and dimer (charge states 24+ to 28+), p53 monomer (charge states 12+ to 15+ with zero, one, two, or three acetylations), and the $^{Ac}p53_{L344P}$:Sirt1 complex (1:1) (charge states 21+ to 24+) have been detected.

Figure S8: Native MS confirms acetylation-dependent complex formation between $p53_{L344A}$ and Sirt1. (A) For non-acetylated $p53_{L344A}$, Sirt1 monomer (charge states 14+ to 19+) as well as $p53_{L344A}$ monomer (charge states 11+ to 14+) and $p53_{L344A}$ dimer (charge states 16+ to 20+) were observed. (B) For $^{Ac}p53_{L344A}$, Sirt1 monomer (charge states 14+ to 18+) and dimer (charge states 23+ to 26+), $p53_{L344A}$ monomer (charge states 11+ to 14+), $p53_{L344A}$ dimer (charge states 17+ to 20+), and the $^{Ac}p53_{L344A}$:Sirt1 complex (1:1) (charge states 24+ to 27+) have been detected. (C) Collisional activation (CID-MS/MS) of the 27+ charge state of this complex (orange box in B) resulted in the ejection of a $^{Ac}p53_{L344A}$ monomer (charge states 15+ to 20+, m/z ~2 000 to ~3 000) from the complex. The remaining $p53_{L344A}$:Sirt1 (charge states 9+ to 12+, m/z ~10 000 to ~13 300) was observed as well.

Figure S9: Native MS confirms acetylation-dependent complex formation between $p53_{L344A}$ and Sirt1. For $^{Ac}p53_{L344A}$, Sirt1 monomer (charge states 14+ to 18+) and dimer (charge states 23+ to 26+), $p53_{L344A}$ monomer (charge states 11+ to 14+ with zero, one, or two acetylations), $p53_{L344A}$ dimer (charge states 17+ to 20+), and the $^{Ac}p53_{L344A}$:Sirt1 complex (1:1) (charge states 24+ to 27+) have been detected.

Figure S10: Fragment ion mass spectra of intermolecular cross-linked products between p53 and Sirt1 with DSBU, automatically annotated by MeroX, b- and y-type ions are shown in blue and red*;* fragment ions of the cross-linker are shown in yellow. Amino acid sequences of the cross-linked peptides are shown on the right, with observed fragments annotated and color-coded according to their relative intensity, B (in the amino acid sequence) indicates carbamidomethylated Cys. $[M + 4H]^{4+}$ at *m/z* 775.433, K382 of p53 cross-linked to K427 of Sirt1.

Figure S11: Fragment ion mass spectra of intermolecular cross-linked products between p53 and Sirt1 with DSBU, automatically annotated by MeroX, b- and y-type ions are shown in blue and red*;* fragment ions of the cross-linker are shown in yellow. Amino acid sequences of the cross-linked peptides are shown on the right, with observed fragments annotated and color-coded according to their relative intensity, B (in the amino acid sequence) indicates carbamidomethylated Cys. $[M + 4H]^{4+}$ at *m/z* 852.686, K120 of p53 cross-linked to K622 of Sirt1.

Figure S12: AlphaFold2 model preparation for molecular docking. Atomic models are colored according to the plDDT confidence score (see color bar). Both initial models were retrieved from AlphaFold-DB. Low-confidence regions outside of folded domains were removed to allow for rigid-body docking. (A) p53. From the AlphaFold2 model, the $p53_{DBD}$ consists of residues 95–295, $p53_{TET}$ comprises residues 320–360. (B) Sirt1. The core region of Sirt1 (residues 183–670), with residues 501–633 missing, were selected and used for rigid-body docking.

Figure S13: Superimposition of the docked p53*:*Sirt1 complex on the $p53_{TET}$ crystal structure. Four 1:1 complexes of p53*:*Sirt1 were aligned on the published $p53_{TET}$ structure (PDB 1c26). There is no clash between the ordered domains, but the $p53_{DBD}$ structures are disconnected by 84-114 Å. The first rotation is 90° at the y-axis, the second rotation 90° at the z-axis (green: $p53_{TET}$; purple: $p53_{DBD}$; salmon: Sirt1).
