# Supplementary material for: Revisiting the p53:Sirt1 interaction in light of controlling p53 acetylation levels
Source: Commun Chem. 2026 Jul 10;9:243. doi: 10.1038/s42004-026-02127-y (PMC13354560; doi:10.1038/s42004-026-02127-y)
Supplement: Supplementary file 2 — Description of Additional Supplementary Files [file 42004_2026_2127_MOESM2_ESM.pdf]

Description of Additional Supplementary Files:

File: Supplementary Data 1

Description: Peptides crosslinked between p53 and Sirt1

File: Supplementary Data 2

Description: Distance restraints for molecular docking. Segid A corresponds to p53 DNA binding domain, Segid B to Sirt1, and Segid C to the tetramerization domain of p53
