# Supplementary material for: Revisiting the p53:Sirt1 interaction in light of controlling p53 acetylation levels
Source: Commun Chem. 2026 Jul 10;9:243. doi: 10.1038/s42004-026-02127-y (PMC13354560; doi:10.1038/s42004-026-02127-y)
Supplement: Supplementary file 3 — Supplementary Data 1 [file 42004_2026_2127_MOESM3_ESM.pdf]

Supplementary Data 1

Peptides crosslinked between p53 and Sirt1

**p53$_{L344P}$**		**Sirt1**	
Crosslinked site (Residue)	Peptide	Crosslinked site (Residue)	Peptide
320 (K)	320-333 ***K***KPLDGEYFTLQIR	622 (K)	622-627 ***K***BWPNR
321 (K)	320-333 K***K***PLDGEYFTLQIR	622 (K)	622-627 ***K***BWPNR
120 (K)	111-132 LGFLHSGTA***K***SVTBTYSPALNK	622 (K)	622-627 ***K***BWPNR
120 (K)	111-132 LGFLHSGTA***K***SVTBTYSPALNK	561 (K)	557-563 DQAA***K***SN
382 (K)	382-386 ***K***LMFK	427 (K)	425-444 AM***K***YDKDEVDLLIVIGSSLK
357 (K)	352-363 DAQAG***K***EPGGSR	427 (K)	425-444 AM***K***YDKDEVDLLIVIGSSLK
120 (K)	111-132 LGFLHSGTA***K***SVTBTYSPALNK	377 (K)	376-384 Y***K***VDBEAVR
386 (K)	383-393 LMF***K***TEGPDSD	304 (K)	304-311 ***K***DPRPFFK
120 (K)	111-132 LGFLHSGTA***K***SVTBTYSPALNK	254 (K)	249-255 LLQEB***K***K
357 (K)	352-363 DAQAG***K***EPGGSR	233 (K)	217-234 DMTLWQIVINILSEPP***K***R
p53$_{L344A}$		Sirt1	
Crosslinked site (Residue)	Peptide	Crosslinked site (Residue)	Peptide
120 (K)	111-132 LGFLHSGTA***K***SVTBTYSPALNK	622 (K)	622-627 ***K***BWPNR
121 (S)	111-132 LGFLHSGTAK***S***VTBTYSPALNK	622 (K)	622-627 ***K***BWPNR
357 (K)	352-363 DAQAG***K***EPGGSR	427 (K)	425-444 AM***K***YDKDEVDLLIVIGSSLK

382 (K)	382-386 ***K*** LMFK	427 (K)	425-444 AM***K*** YDKDEVDLLIVIGSSLK
373 (K)	373-379 ***K*** GQSTSR	233 (K)	217-234 DMTLWQIVINILSEPP***K*** R
p53$_{wt}$		Sirt1	
Crosslinked site (Residue)	Peptide	Crosslinked site (Residue)	Peptide
120 (K)	111-132 LGFLHSGTA***K*** SVTBTYSPALNK	622 (K)	622-627 ***K*** BWPNR
125 (T)	111-132 LGFLHSGTAKSVTB***T*** YSPALNK	622 (K)	622-627 ***K*** BWPNR
373 (K)	373-379 ***K*** GQSTSR	428 (Y)	425-444 AMK***Y*** DKDEVDLLIVIGSSLK
382 (K)	382-386 ***K*** LMFK	427 (K)	425-444 AM***K*** YDKDEVDLLIVIGSSLK
120 (K)	111-132 LGFLHSGTA***K*** SVTBTYSPALNK	377 (K)	376-384 Y***K*** VDBEAVR
120 (K)	111-132 LGFLHSGTA***K*** SVTBTYSPALNK	238 (K)	238-244 ***K*** DINTIE
123 (T)	111-132 LGFLHSGTAKSV***T*** BTYSPALNK	238 (K)	237-244 R***K*** DINTIE

B is for carbamidomethylated Cys residues

Sirt1 intra-molecular crosslinks and dead-end crosslinks obtained from LC-MS/MS analysis of in-solution digestion of $^{Ac}p53_{L344P}$ +Sirt1 + DSBU crosslinking reactions

Sirt1 intra-protein crosslinks	site 1	residue	site 2	residue
1	513	K	610	K
2	248	K	506	K
3	248	K	508	S
4	513	K	517	T
5	513	K	519	K
6	506	K	630	K
7	238	K	535	S
8	517	T	734	K
9	519	K	734	K
10	622	K	630	K
11	238	K	517	T
12	238	K	519	K
13	614	T	622	K
14	615	S	622	K
15	513	K	622	K
16	338	K	376	Y
17	338	K	377	K
18	200/203	T/K	630	K
19	535	S	630	K
20	571/572	S/K	630	K
21	601	K	614/615	T/S
22	254/255	K/K	335/338	K/K
23	200/203	T/K	238	K
24	519	K	738	T
25	304	K	732/734	S/K
26	513	K	630	K
27	506	K	622	K
28	630	K	517/519	T/K
29	200/203	T/K	427/428/430	K/Y/K
30	506	K	584/585	T/S
31	335	K	499	K
32	499	K	622	K
33	203	K	535	S
34	622	K	517/519	T/K
35	314	K	622	K
36	519	K	544	T
37	517/519	T/K	200/203	T/K

38	314	K	630	K
39	233	K	430	K
40	233	K	428	Y
41	314	K	610	K
42	233	K	236/238	K/K
43	427/428/430	K/Y/K	561/562	K/S
44	427	K	732/734/738	S/K/T
45	233	K	610	K
46	370	S	377	K
47	238	K	248	K
48	561/562	K/S	314	K
49	338	K	248	K
50	427	K	254	K
51	427	K	376/377	Y/K
52	233	K	630	K
53	571/572	S/K	561/562	K/S
54	238	K	610	K
55	335	K	506	K
56	578	K	233	K
57	519	K	530/535	T/S
58	242	T	248	K
59	427/428	K/Y	610	K
60	233	K	622	K
61	238	K	622	K
62	622	K	610	K
63	314	K	734	K
64	314	K	377	K
65	578	K	601	K
66	622	K	658/659	Y/S
67	238/242	K	254/255	K
68	314	K	601	K
69	610	K	517/519	T/K
70	630	K	746/747	K/S
71	304	K	314	K
72	233	K	561	K
73	376/377	Y/K	408	K
74	233/235	K/K	254/255	K/K
75	233	K	615	S
76	376	Y	572	K
77	376/377	Y/K	622	K
78	622	K	744/746	S/K

**Sirt1 dead-end crosslinks**	**site**	**residue**
1	517	T
2	519	K
3	427	K
4	255	K
5	375	K
6	376	Y
7	377	K
8	430	K
9	506	K
10	630	K
11	233	K
12	235	K

Sirt1 intra-molecular crosslinks and dead-end crosslinks obtained from LC-MS/MS analysis of in-gel digestion of excised $^{Ac}p53_{L344P}$ + Sirt1 + DSBU crosslinked band

Sirt1 intra-protein crosslinks	site 1	residue	site 2	residue
1	513	K	610	K
2	513	K	519	K
3	513	K	517	T
4	622	K	630	K
5	238	K	517	T
6	238	K	519	K
7	614	T	622	K
8	615	S	622	K
9	601	K	614/615	T/S
10	254/255	K/K	335/338	K/K
11	304	K	732/734	S/K
12	513	K	630	K
13	630	K	517/519	T/K
14	335	K	499	K
15	233	K	236/238	K/K
16	238	K	248	K
17	571/572	S/K	561/562	K/S
18	238	K	610	K
19	519	K	530/535	T/S
20	622	K	610	K
21	578	K	601	K
22	622	K	658/659	Y/S
23	238/242	K	254/255	K
24	630	K	658/659	Y/S
25	630	K	335/338	K
26	513	K	601	K
27	630	K	642	K
28	610	K	304	K
29	304	K	630	K
30	601	K	561	K
31	601	K	622	K
32	614	T	630	K
33	530	T/K	561	K
34	513	K	335	K
35	601	K	517/519	T/K
36	630	K	561/562	K/S
37	535	S	561	K
38	517/519	T/K	561	K

39	630	K	610	K
40	506/508/511	K/S/T	517/519	T/K
41	734	K	744/746/747	S/K/S
42	601	K	630	K
43	513	K	236/238	K/K
44	610	K	561	K
45	734	K	753	K
46	561	K	578	K
47	610	K	614/615/619	T/S

**Sirt1 dead-end crosslinks**	**site**	**residue**
1	517	T
2	519	K
3	427	K
4	375	K
5	376	Y
6	377	K
7	630	K
8	233	K
9	235	K
10	642	Y
11	746	S
12	747	S
13	169	S
14	314	K
15	453	S
16	454	S
17	172	S
18	173	S
19	174	S
20	441	S
21	442	S
22	444	K
23	506	K
24	571	S
25	572	K

Sirt1 intra-molecular crosslinks and dead-end crosslinks obtained from LC-MS/MS analysis of in-solution digestion of Sirt1 + DSBU crosslinking reaction

**Sirt1 intra-protein crosslinks**	**site 1**	**residue**	**site 2**	**residue**
1	622	K	630	K
2	238/242	K	254/255	K
3	734	K	744/746/747	S/K/S
4	572	K	569	S
5	578	K	569	S
6	368	T	377	K
7	370	S	377	K
8	513	K	506	K
9	513	K	508	S

**Sirt1 dead-end crosslinks**	**site**	**residue**
1	375	K
2	376	Y
3	377	K
4	506	K
5	630	K
6	746	S
7	747	S
8	571	S
9	572	K
10	578	K
11	622	K
12	610	K

$^{Ac}p53_{L344P}$ intra-molecular crosslinks and dead-end crosslinks obtained from LC-MS/MS analysis of in-solution digestion of $^{Ac}p53_{L344P}$ + Sirt1 + DSBU crosslinking

$^{Ac}p53_{L344P}$ intra-protein crosslinks	site 1	residue	site 2	residue
1	319	K	321	K
2	163/164/166/170	Y/K/S/T	291/292	K/K
3	319/320/321	K/K/K	357	K
4	321	K	327	Y
5	163/164	Y/K	357	K
6	370/371/372	K/S/K	373	K
7	357	K	370/371/372/373	K/S/K/K
8	366/367	S/S	373	K
9	370/371/372/373	K/S/K/K	381	K
10	118/120	T/K	381/382/386	K/K/K
11	118/120/121/123	T/K/S/T	139/140	K/T
12	118/120/121	K	320/321	K/K
13	319/320/321	K/K/K	381/382	K/K
14	102	T	164	K
15	163/164	Y/K	382	K
16	24	K	319/320/321	K/K/K
17	139/140	K/T	381/382/386	K/K/K
18	139	K	149	S
19	320/321	K/K	163/164	Y/K
20	24	K	116/118/120/121	S/T/K/S
21	24	K	357	K
22	321	K	357	K
23	139/140	K/T	227	S
24	24	K	382	K
25	24	K	164	K
26	321	K	381	K
27	373	K	320	K
28	166/170	S/T	291	K
29	24	K	386	K

30	24	K	303/304/305	S/T/K
31	118/120/121	T/K/S	351/357	K/K
32	139	K	240	S
33	320/321	K/K	351	K
34	240/241	S/S	120	K
35	118/120/121	T/K/S	164	K
36	319/320/321	K/K/K	313/314/315	S/S/S
37	357	K	139	K
38	118/120/121	T/K/S	319/320/321	K/K/K
39	164	K	303/304/305	S/T/K
40	163	Y	373	K
41	320	K	386	K
42	304	T	320	K
43	362	S	370	K
44	367	K	320/321	K/K
45	319	K	327	Y
46	373	K	362	S
47	120	K	370	K
48	24	K	291/292	K/K
49	319/320	K/K	139/140	K/T
50	15/20	S/S	24	K
51	351	K	381	K
52	305	K	381	K
53	0	N-term/S	320	K
54	164	K	351	K

^{Ac}**p53$_{L344P}$** **dead-end crosslinks**	**site**	**residue**
1	260	S
2	164	K
3	116	S
4	118	T
5	120	K
6	139	K
7	140	T
8	240	S
9	241	S
10	319	K
11	320	K
12	321	K

^{Ac}p53$_{L344P}$ intra-molecular crosslinks and dead-end crosslinks obtained from LC-MS/MS analysis of in-gel digestion of excised ^{Ac}p53$_{L344P}$ + Sirt1 + DSBU crosslinking reactions

^{Ac}p53$_{L344P}$ intra-protein crosslinks	site 1	residue	site 2	residue
1	319	K	321	K
2	163/164/166/170	Y/K/S/T	291/292	K/K
3	319/320/321	K/K/K	357	K
4	357	K	386	K
5	381	K	386	K
6	321	K	327	Y
7	163/164	Y/K	357	K
8	370/371/372	K/S/K	373	K
9	357	K	370/371/372/373	K/S/K/K
10	366/367	S/S	373	K
11	373	K	386	K
12	357	K	366	K
13	357	K	351	K
14	370/371/372/373	K/S/K/K	381	K

^{Ac}p53$_{L344P}$ dead-end crosslinks	site	residue
1	303	S
2	304	T
3	305	K
4	291	K
5	292	K
6	260	S
7	164	K
8	116	S
9	118	T
10	120	K
11	139	K
12	140	T
13	351	K

14	205	Y
15	357	K
16	240	S
17	241	S
18	319	K
19	320	K
20	321	K

$^{Ac}p53_{L344P}$ intra-molecular crosslinks and dead-end crosslinks obtained from LC-MS/MS analysis of in-solution digestion of $^{Ac}p53_{L344P}$ + DSBU crosslinking reactions

$^{Ac}p53_{L344P}$ intra-protein crosslinks	site 1	residue	site 2	residue
1	381	K	386	K
2	370/371/372	K/S/K	373	K
3	357	K	370/371/372/373	K/S/K/K
4	357	K	351	K
5	319/320/321	K/K/K	313/314/315	S/S/S
6	0	N-term/S	320	K
7	0	N-term/S	164	K
8	0	N-term/S	118/120/121	T/K/S
9	0	N-term/S	382	K
10	366/367	S/S	372	K

$^{Ac}p53_{L344P}$ dead-end crosslinks	site	residue
1	303	S
2	304	T
3	305	K
4	291	K
5	292	K
6	164	K
7	116	S
8	118	T
9	120	K
10	139	K
11	140	T
12	351	K
13	357	K
14	319	K
15	320	K
16	321	K
