# Supplementary material for: Revisiting the p53:Sirt1 interaction in light of controlling p53 acetylation levels
Source: Commun Chem. 2026 Jul 10;9:243. doi: 10.1038/s42004-026-02127-y (PMC13354560; doi:10.1038/s42004-026-02127-y)
Supplement: Supplementary file 4 — Supplementary Data 2 [file 42004_2026_2127_MOESM4_ESM.pdf]

Supplementary Data 2

Distance restraints for molecular docking. Segid A corresponds to p53 DNA binding domain, Segid B to Sirt1, and Segid C to the tetramerization domain of p53

assign (segid A and resid 120 and name CA) (segid B and resid 1254 and name CA) 25 20 0
assign (segid A and resid 120 and name CA) (segid B and resid 1377 and name CA) 25 20 0
assign (segid A and resid 120 and name CA) (segid B and resid 1561 and name CA) 25 20 0
assign (segid A and resid 120 and name CA) (segid B and resid 1622 and name CA) 25 20 0
assign (segid C and resid 320 and name CA) (segid B and resid 1633 and name CA) 30 25 0
assign (segid C and resid 321 and name CA) (segid B and resid 1633 and name CA) 30 25 0
assign (segid C and resid 357 and name CA) (segid B and resid 1233 and name CA) 25 20 0
assign (segid C and resid 357 and name CA) (segid B and resid 1427 and name CA) 25 20 0
assign (segid A and resid 274 and name CA) (segid C and resid 320 and name CA) 30 30 0
assign (segid C and resid 360 and name CA) (segid B and resid 1363 and name CA) 30 30 0
